# Herpes Simplex Virus Hepatitis: A Presentation of Multi-Institutional Cases to Promote Early Diagnosis and Management of the Disease

**DOI:** 10.1155/2017/3180984

**Published:** 2017-08-01

**Authors:** Ashwinee Natu, Guiseppe Iuppa, Clifford D. Packer

**Affiliations:** ^1^Department of Internal Medicine, University Hospitals Cleveland Medical Center, 11100 Euclid Ave., Cleveland, OH 44106, USA; ^2^Department of Transplant Surgery, Cleveland Clinic, 9500 Euclid Ave., Cleveland, OH 44195, USA; ^3^Department of Internal Medicine, Louis Stokes VA Medical Center, 10701 East Blvd., Cleveland, OH 44106, USA

## Abstract

**Objective:**

To compare three cases of Herpes simplex virus (HSV) hepatitis to increase early diagnosis of the disease.* Case*  *1.* A 23-year-old man with Crohn's disease and oral HSV. HSV hepatitis was diagnosed clinically and he improved with acyclovir.* Case*  *2.* An 18-year-old G1P0 woman with transaminitis. Despite early empiric acyclovir therapy, she died due to fulminant liver failure.* Case*  *3*. A 65-year-old woman who developed transaminitis after liver transplant. Diagnosis was confirmed by biopsy and she had resolution of acute liver failure with acyclovir.

**Conclusion:**

It is imperative that clinicians be aware of patients at high risk for developing HSV hepatitis to increase timely diagnosis and prevent morbidity and fatality.

## 1. Introduction

Herpes simplex virus (HSV) hepatitis is a rare diagnosis that can rapidly progress to fulminant liver failure. Timely diagnosis and treatment of this disease are imperative in reducing patient morbidity and mortality. We describe three cases of HSV hepatitis that highlight the commonalities and differences in presentation and discuss keys to early diagnosis.

## 2. Case  1

**Figure 1:**
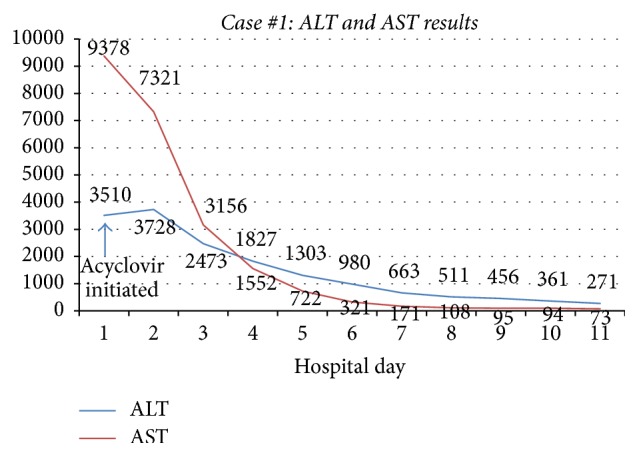
Case #1 trend in ALT and AST. ALT: alanine aminotransferase; AST: aspartate aminotransferase.

**Figure 2:**
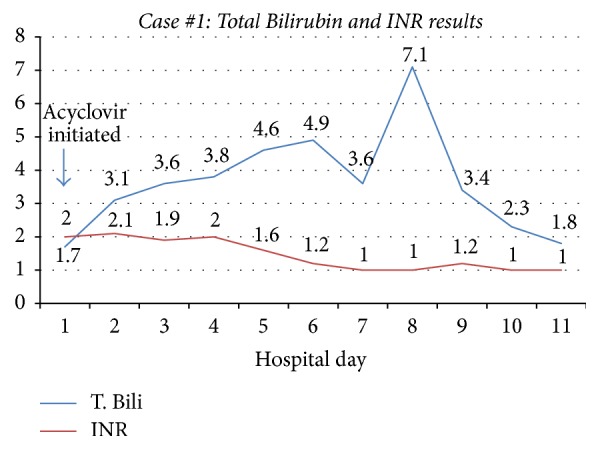
Case #1 trend in Total Bilirubin and INR. T. Bili: Total Bilirubin; INR: International Randomized Ratio.

A 23-year-old man with severe fistulizing Crohn's disease was admitted to the hospital with abdominal pain. Symptoms included right upper quadrant abdominal pain, fever, tachycardia, and increased nonbloody bowel movements. He had been on a prednisone taper and vedolizumab. Initial lab findings were significant for an elevation in aminotransferases (ALT 3510 U/L, AST 9378 U/L) (Figures 1 and 2). Abdominal imaging was negative for acute pathology including vascular liver disease. He was started on broad-spectrum antibiotics, acyclovir, and N-acetylcysteine. Additional testing was notable for positive HSV-2 IgM antibody test, HSV-2 detectability by DNA PCR, and genital/oral cultures positive for HSV-2. Based on these results he was diagnosed with HSV hepatitis and treated with acyclovir. Despite initial clinical improvement, his course was further complicated by a recurrent Crohn's flare. He was eventually discharged on high dose oral acyclovir, budesonide, and prednisone. As his HSV viral titer drastically decreased, he was restarted on outpatient vedolizumab infusions. He was continued on lifelong acyclovir for viral suppression due to his immunosuppression.

## 3. Case  2

**Figure 3:**
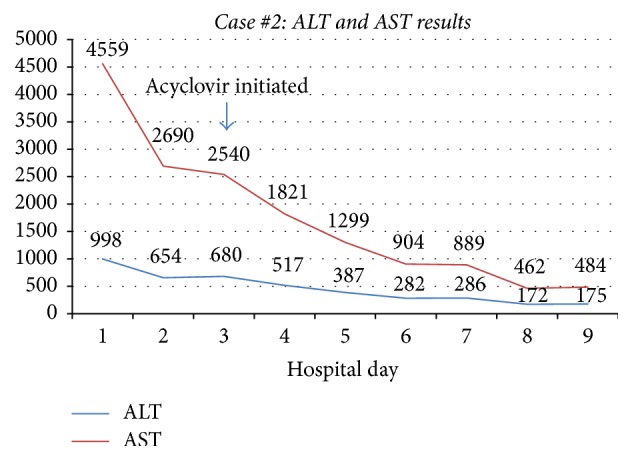
Case #2 trend in AST and ALT. ALT: alanine aminotransferase; AST: aspartate aminotransferase.

**Figure 4:**
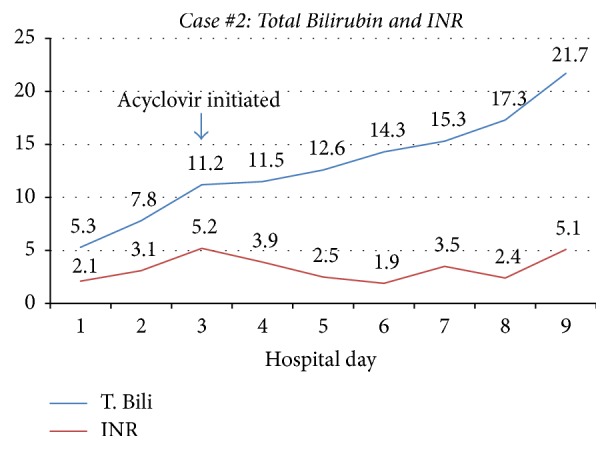
Case #2 trend in Total Bilirubin and INR. T. Bili: Total Bilirubin; INR: International Randomized Ratio.

An 18-year-old G1P0 woman with no significant past medical history presented to an outside hospital at 26-week gestation with shortness of breath. On presentation, she was febrile and tachycardic with laboratory data significant for aminotransferase elevation (ALT 998 U/L, AST 4559 U/L) and disseminated intravascular coagulopathy (DIC) (Figures 3 and 4). She underwent emergent cesarean section due to chorioamnionitis and a male infant was delivered. By day 2, she developed ascites and encephalopathy. A CT scan showed liver findings consistent with fatty infiltration and hemorrhage. She was transferred to our institution for transplant evaluation due to acute liver failure from presumed acute fatty liver of pregnancy. Upon transfer, she required intubation for airway protection and laboratory data revealed worsening multiorgan compromise. Antibiotics were broadened, and empiric acyclovir was initiated on day 3. Continuous venovenous hemofiltration was initiated for management of anasarca and anuria. On day 4 of transfer, her infant reportedly died of HSV-2 sepsis at another hospital. On day 6, our transplant committee determined she was not a transplant candidate due to profound psychosocial concerns. The patient's HSV-2 IgM antibody test and HSV-2 DNA by PCR returned positive and repeat blood cultures grew candida albicans. She subsequently developed septic shock requiring vasopressor support and her family decided on no further escalation of care. On day 9, she died from complications of fulminant liver failure.

## 4. Case  3

**Figure 5:**
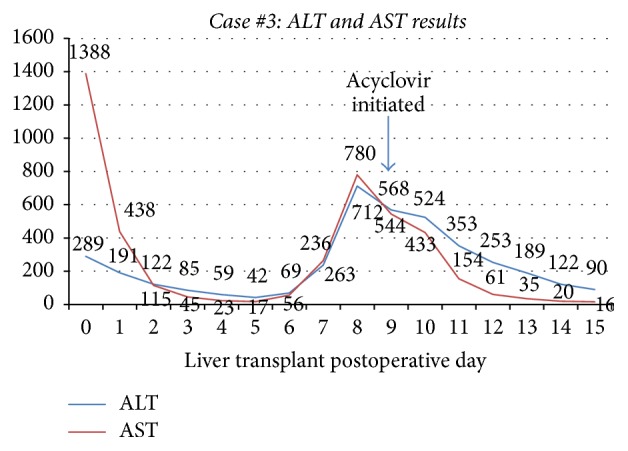
Case #3 trend in AST and ALT. ALT: alanine aminotransferase; AST: aspartate aminotransferase.

**Figure 6:**
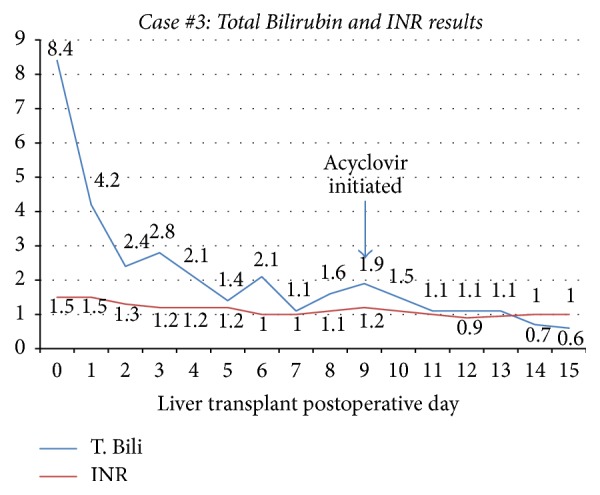
Case #3 trend in Total Bilirubin and INR. T. Bili: Total Bilirubin; INR: International Randomized Ratio.

**Figure 7:**
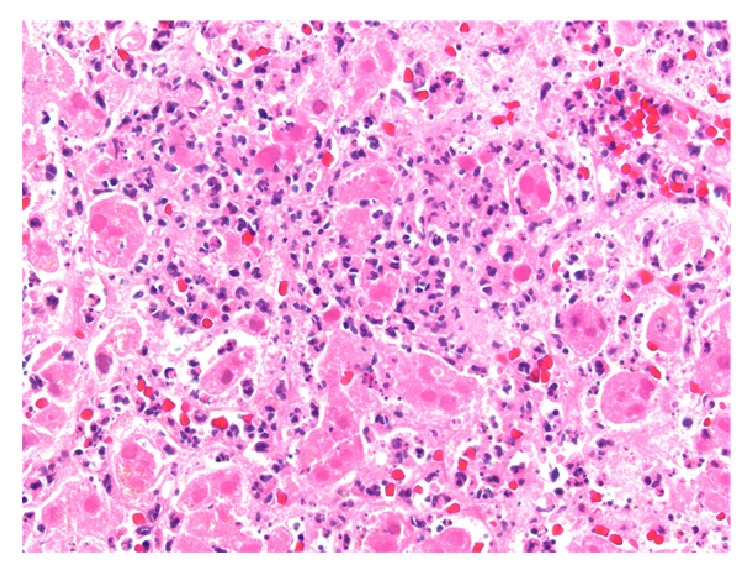
Liver biopsy from patient 3 showing intranuclear viral inclusions within the necrotic areas.

**Figure 8:**
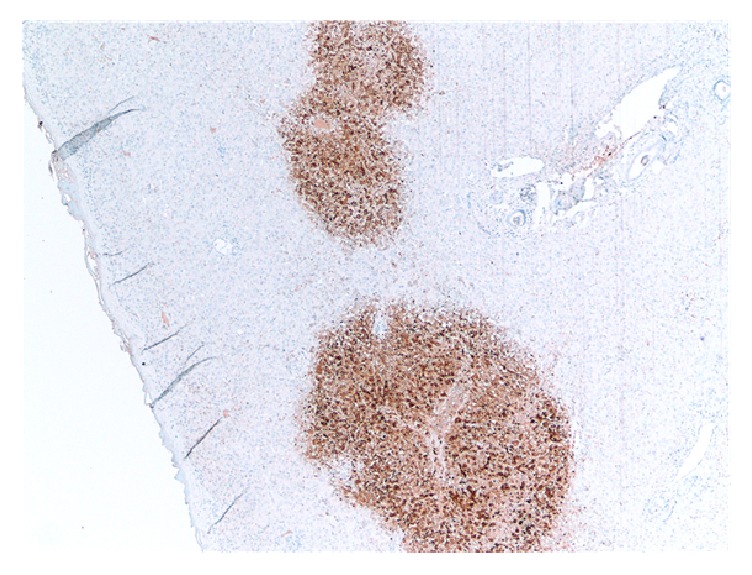
HSV immunostain from liver biopsy (patient 3).

A 65-year-old woman with cirrhosis due to alcohol abuse complicated by refractory hydrothorax and ascites was admitted to the hospital with worsening dyspnea due to hydrothorax. She subsequently underwent uncomplicated liver transplantation with a donor liver from a 29-year-old woman that was IgG antibody positive for Epstein Barr Virus and Cytomegalovirus. She was initiated on immunosuppressive therapy with tacrolimus, mycophenolate mofetil, and a prednisone taper along with prophylactic valganciclovir. Her immediate postoperative course was uncomplicated and liver function tests remained stable. On postoperative day (POD) 8, she had an acute increase in aminotransferases (ALT of 712 U/L, AST of 780 U/L) with all other values within normal limits (Figures 5 and 6); liver ultrasound did not show abnormalities. She complained of worsening abdominal pain as her aminotransferases continued to uptrend and she was started on broad-spectrum antibiotics. CT scan of the abdomen was significant for suspected mesenteric artery pseudoaneurysm not present in past studies. She underwent laparotomy for removal of the aneurysm and liver biopsy on POD 8 that showed patchy areas of hepatocyte necrosis (Figures 7 and 8). At this time, her serum HSV-2 IgM antibody and HSV DNA by PCR returned positive and she was initiated on intravenous acyclovir. Of note, she denied prior history of oral or genital HSV infection. Pathology eventually returned as HSV hepatitis with hepatic necrosis. She had complete resolution of transaminase elevation within 5 days of treatment initiation and had an uncomplicated recovery.

## 5. Discussion and Literature Review

HSV hepatitis is an uncommon cause of acute liver failure, accounting for 0.8% of all cases and only 2% of all viral hepatitis [1, 2]. It is mostly seen in immunocompromised individuals and pregnant women in their third trimester following an orogenital HSV-1 or HSV-2 infection, though previous reports have shown up to 25% of cases in immunocompetent individuals [1]. Due to lack of specific clinical findings, the diagnosis is frequently missed on presentation and can lead to rapid progression to fulminant liver failure and multiorgan collapse if untreated [3]. The patients in all three cases are typical of the population at high risk for HSV hepatitis.

Liver biopsy remains the only gold standard for diagnosis of HSV hepatitis. However the procedure is often not feasible due to coagulopathy or ascites, and it is often necessary to make the diagnosis based on laboratory testing and symptoms [1]. Only the patient in case  3 underwent biopsy that showed histology pathognomonic for HSV infection: hemorrhagic necrosis, inflammation, enlarged ground glass nuclei with marginalized chromatin, and HSV+ immunostaining [4].

Of the presented cases, only the patient in case  1 had oral and genital mucocutaneous lesions, which, while useful in supporting the diagnosis, are absent in up to 50% of cases [3, 5]. Serologic testing is commonly done but carries a high rate of false-negativity and therefore should be used in conjunction with confirmatory PCR testing. All three of the above patients had evidence of disseminated disease with high levels of HSV DNA, and in cases 1 and 3, viral titers dropped after initiation of acyclovir.

Other common features of HSV hepatitis in the absence of a biopsy are aminotransferases >500, fever, coagulopathy, encephalopathy, leukopenia, thrombocytopenia, and acute renal failure (ARF) [2, 4, 6] (see Table 1 for a summary of patient data and Table 2 for epidemiologic data from two large literature reviews on HSV hepatitis) [1, 7]. While all of these were evident in cases  1 and 2, the patient in case  3 had a solitary rise in aminotransferases and mild ARF. Additionally, previous reports have noted an anicteric pattern of hepatitis as a commonality in presentation of this disease, though this was not seen in case  1 or 2 in this series [2, 8]. This suggests that these findings and patterns together may support a diagnosis of HSV hepatitis but lack sensitivity and should not be used to rule out the diagnosis.

Previous cases have shown that early initiation of acyclovir (usually within 3 days of presentation) leads to better outcomes due to the cessation of viral replication and spread of infection [1, 6, 8]. While the patients in case  1 and 3 had near complete resolution of hepatitis within 5 days of initiation of acyclovir, unfortunately case  2 had rapid progression to fulminant hepatic failure and death, possibly because her disease burden was already too great on presentation or that the virus was acyclovir resistant. Though data is sparse, intravenous foscarnet is the agent of choice in cases of acyclovir-resistant herpetic infection; however even dual-agent therapy has proven ineffective in preventing mortality in previous case reports [9]. In general, the data also suggest that acyclovir should be started empirically for high-risk patients with ALF of unknown etiology, pending laboratory confirmation of the diagnosis. However, prospective data on empiric therapy in specific populations are lacking.

In conclusion, there should be a high suspicion for HSV hepatitis as a cause for fulminant liver failure, especially in high-risk patient populations. Though diagnosis with biopsy is the gold standard, HSV PCR with concurrent elevation in aminotransferases can serve as substitute markers for making the diagnosis. HSV hepatitis should not be excluded based on the lack of mucocutaneous lesions, systemic inflammatory reaction markers, ARF, or anicteric hepatitis; however these findings may be used to support a suspected diagnosis. Given the favorable side effect profile of acyclovir, empiric treatment should be initiated early in presentation.

## Figures and Tables

**Table 1:** Peak/nadir labs and outcomes of patients presented.

	Predisposing factor	AST (U/L)	ALT (U/L)	Bilirubin (mg/dL)	Platelets (×10^3^/*μ*L)^4^	INR	GFR (mL/min/m^2^)	Outcome
Case 1	Crohn's- immunosuppression, steroids	9378	3510	7.4	130	2.1	42	Survived
Case 2	Pregnancy (2nd trimester)	4559	998	21.7	33	5.1	30	Died
Case 3	Liver transplant- immunosuppression, steroids	780	712	1.9	107	1.3	96	Survived

ALT: alanine aminotransferase; AST: aspartate aminotransferase; INR: international normalized ratio; GFR: glomerular filtration rate.

**Table 2:** Epidemiology based on two large literature reviews.

	Norvell et al.	Kaufman et al.
	Number (%)	Number (%)
*N*	137	52
Age (mean, yrs)	34	N/A
Gender		N/A
(i) Male	51/137 (38)	
(ii) Female	86/137 (62)	
Immunosuppressed	72/137 (53)	35/52 (67)
Pregnant	32/137 (23)	9/52 (17)
Fever	98/100 (98)	42/52 (82)
Herpetic lesions	54/123 (44)	29/52 (57)
Mean peak ALT or AST	4927	N/A
Leukopenia	50/70 (71)	22/52 (43)
Thrombocytopenia	59/63 (93)	23/52 (45)
Coagulopathy	93/111 (83)	10/52 (20)
Acute Renal Failure	34/52 (65)	N/A
Death		
(i) Acyclovir	25/49 (51)	5/9 (55)
(ii) No Acyclovir	74/84 (88)	34/43 (80)

ALT: alanine aminotransferase; AST: aspartate aminotransferase; N/A: not available.
